# Care needs of Japanese men for sexual dysfunction associated with prostate cancer treatment

**DOI:** 10.1007/s00520-023-07837-w

**Published:** 2023-06-05

**Authors:** Saeko Hayashi, Kazuki Sato, Fumiko Oishi, Hiromi Fukuda, Yuka Hayama, Shoko Ando

**Affiliations:** 1 Nagoya University Graduate School of Medicine, Nagoya, Japan; 2 Aichi Medical University, Nagakute, Aichi Japan; 3 School of Nursing, Seirei Christopher University, Shizuoka, Japan; 4 Aichi University of Education, Kariya, Aichi Japan; 5 Doshisha Women’s College of Liberal Arts, Kyoto, Japan; 6 School of Nursing, Ichinomiya Kenshin College, Ichinomiya, Aichi Japan

**Keywords:** Care needs, Sexual dysfunction, Prostate cancer treatment, Japanese men, Qualitative research

## Abstract

**Purpose:**

Prostate cancer (PC) treatment causes sexual dysfunction (SD) and alters fertility, male identity, and intimate relationships with partners. In Japan, little attention has been paid to the importance of providing care for SD associated with PC treatment. This study is aimed at clarifying the care needs of Japanese men regarding SD associated with PC treatment.

**Methods:**

One-to-one semi-structured interviews were conducted with 44 PC patients to identify their care needs. Data were analyzed using thematic analysis.

**Results:**

Four core categories emerged from the analysis. (1) “Need for empathy from medical staff regarding fear of SD”: patients had difficulty confiding in others about their sexual problems, and medical staff involvement in their SD issues was lacking. (2) “Need for information that provides an accurate understanding of SD and coping strategies before deciding on treatment”: lack of information about SD in daily life and difficulty understanding information from medical institutions, caused men to regret their treatment. (3) “Need for professional care for individuals and couples affected by SD”: men faced loss of intimacy because of their partners’ unwillingness to understand their SD issues or tolerate non-sexual relationships. (4) “Need for an environment that facilitates interaction among men to resolve SD issues”: men felt lonely and wanted to interact with other patients about their SD concerns.

**Conclusion:**

These findings may help form care strategies tailored to these needs and applicable to other societies with strong traditional gender norms.

## Background

Prostate cancer (PC) is the most common type of cancer in men worldwide [1]. Most patients are in their 60s or older, but in recent years, the number of patients in their 40s has been increasing. Japanese patients’ 5-year survival rate from PC is better than that from other carcinomas [2, 3]. As PC treatment ensures a long post-treatment life expectancy, managing side effects is crucial.

PC treatment reduces men’s sexual function and alters their fertility, masculine identity, and relationships with their partners, leading to distress [4, 5]. Care for SD associated with PC treatment has been studied in other countries that provide pharmacotherapy, erectile aids, and psychological support [6, 7]. Recently, self-compassion [8] was shown to alleviate gender-role conflict and distress in PC patients [9]; experts have also examined mindfulness interventions [10]. Many couples recognize and address the importance of communication and the appropriate use of erectile aids and medications to maintain sexual intimacy and a positive attitude toward each other. These trends influence the evolution of their identity as a couple [11].

However, few studies have examined this issue in Japan, where care is insufficient [12, 13]. Physicians rarely attend educational workshops on cancer and sexuality for healthcare providers [14]. Moreover, few nurses are experienced in providing care for PC patients [12]. Consequently, PC patients cope with SD by self-medicating or by changing their cognitions. Some use erectile drugs purchased over the Internet or interrupt treatment at one’s discretion, leading to the deterioration of health. Some decide that their partners do not need sex, leading to separation from the partner [5]. Japanese individuals generally do not publicly complain about sexual problems [15, 16]. Further, many patients lived through the postwar reconstruction period and grew up honoring *silent masculinity*, which expects men to endure their suffering alone rather than seek help [17]. This makes it difficult for healthcare professionals to identify patients’ care needs. This research is aimed at identifying and developing a deep understanding of PC patients’ needs regarding SD, including the types of care for SD associated with PC treatment from patients’ perspectives. Our research question was “What are Japanese men’s care needs regarding SD-related to PC treatment?”

## Methods

### Study design and participants

SD associated with PC treatment care needs are guided by individuals’ complex and diverse backgrounds. We used a qualitative inductive research design [18] wherein the complex phenomenon was studied in its natural everyday context, to discover new aspects and generate effective and flexible theories regarding diverse and complex issues.

Participant selection criteria included Japanese men with PC who consented to participate. Exclusion criteria were physical or mental inability to be interviewed and being younger than 20 years. Sampling was conducted with consideration given to population diversity, including various treatment options; a wide range of ages; the full range of married, unmarried, partnered, divorced, or widowed men; various social roles; and urban and rural residents. Sampling was terminated when we reached data saturation and could confirm that no new information would be obtained from additional data.

### Procedures

We recruited participants from PC patient associations nationwide and from five hospitals/clinics in urban and rural areas. Research cooperation request forms with the researchers’ contact information were placed at each facility. One-on-one participant interviews were conducted in private conference rooms where privacy could be ensured.

Before the interview, the investigator explained the study purpose and procedures and notified individuals that the interviews would be recorded and that anonymous quotations from the interviews would be included in published results. Volunteers who agreed to participate provided written informed consent. The interviews focused on situations that prompted participants to consider their SD care needs (Table 1).

**Table 1:** Interview guide

What kind of support do you think is needed for sexual dysfunction associated with prostate cancer treatment?	
Please share the incidents that led you to this conclusion.	

The interviewer was a middle-aged woman researcher who had attended the Sex Counseling Training Course of the Japan Society for Sexology. The interviewer treated sexuality as a normal part of life and did not judge interviewees on the basis of her values; instead, she motivated them to speak freely and as precisely as possible to provide an accurate picture of their experiences. The interviewer summarized or repeated what she heard to confirm her understanding and focused on interviewees’ emotional dynamics. The interviews, transcriptions, and data analyses were conducted in Japanese to obtain comprehensive narratives. The principal researcher managed recruitment, informed consent, interviews, and initial analysis. Participants were identified with numbers instead of names to ensure privacy. Interviews were conducted from February 2019 to November 2020. Recordings and transcripts remain on a password-protected computer for the study, which complies with the Consolidated Standards for Reporting of Qualitative Research (COREQ) [19].

We conducted qualitative content analysis on the interview data [20] and engaged in thorough discussions until we reached a consensus on the themes that emerged. Researchers explained the research methodology to interviewees and described the categories in detail, quoting interviewees when necessary. Several interviewees confirmed the data integrity, and all were satisfied with the relevance of the data.

## Results

### Participant characteristics

Forty-eight individuals participated; four were excluded on the basis of selection and exclusion criteria. Ten of the remaining 44 participants received radical prostatectomy and 13 received external-beam radiation therapy (EBRT); five received brachytherapy (LDR); 12 had combined androgen blockade (CAB); three received combined treatment; and one was in watchful waiting (Table 2). Ages at diagnosis ranged from 47 to 82, with a median of 66.0. Participants described changes in sexual function related to treatment, such as decreased libido, no or poor erection, no or reduced semen, tender orgasm, changed semen properties, ejaculatory pain, or discomfort.

**Table 2:** Demographics of participants (*n* = 44)

Initial treatment	Prostatectomy	10
	EBRT	13
	LDR	5
	CAB	12
	Combined treatment	3
	Watchful waiting	1
Age at diagnosis	Median (range)	66.0 (47–82 years)
Age at survey	Median (range)	71.5 (50–86 years)
Marital status	Married	38
	Unmarried	3
	Divorced	2
Parenting experience	Yes	37
	No	6
Job at diagnosis	Business owner	7
	Employee	23
	Farmer	1
	Part-time job	4
	Retired	8
Sexual intercourse at diagnosis	Active	25
Sexual intercourse at survey	Active	9
Changes in sexual function experienced (participant description and number of participants described)	Decreased libido	9
	None or poor erection	27
	None or decrease in semen	32
	Changes in semen properties	9
	Tender orgasm	13
	Ejaculatory pain or discomfort	3
Medical history	Diabetes	2
	High blood pressure	19
	Heart disease	5
	Chronic kidney disease	2

*EBRT*, external beam radiation therapy; *LDR*, brachytherapy; *CAB*, combined androgen blockade

### Care needs of Japanese men for sexual dysfunction associated with PC treatment

The following themes emerged from participants’ care need narratives: “need for empathy from medical staff regarding fear of SD”; “need for information that provides an accurate understanding of SD and coping strategies before deciding on treatments”; “need for professional care for individuals and couples with SD”; and “need for an environment that facilitates interaction among men to resolve SD issues” (Table 3).

**Table 3:** Themes from the care needs narratives of Japanese men with sexual dysfunction associated with prostate cancer treatment

1. Need for empathy from medical staff regarding fear of sexual dysfunction	
1) Difficulty in confessing sexual unease	
It is difficult for patients to confide sexual problems because of their desire “to keep sexual matters secret” and “not to be obsessed with sex as men with cancer or old adults.”	
Men feel that their desire to attract the opposite sex and maintain sexual function as seniors is unacceptable for medical staff and leads to rumors and disrespect.	
Patients are reluctant to bother busy staff with topics related to sexual functioning, they feel their self-esteem wavering if they confide in staff of the same sex, and they are confused and hesitant to approach staff of the opposite sex.	
2) Lack of staff’s concern about sexual dysfunction resulting from therapy	
Facing the mood and attitude of the staff who appear to disregard the patient’s complaints of sexual dysfunction	
Failure of the staff to strike a conversation or provide information about changes in sexual function and accompanying anxieties	
3) Expectations of involvement from the staff, with empathy for the values of men and the power to persuade them to express worries about sexual dysfunction	
It is necessary to build trust between men and staff so that men can seek substantial advice on sexual dysfunction.	
Men expect the staff to show empathy for each individual based on an understanding of their and their partner’s sexual values.	
Men want the staff to step in and sound out sexuality problems that are difficult to express.	
2. Need for information that provides an accurate understanding of sexual dysfunction and coping strategies before deciding on treatments	
1) Insufficient information on sexual dysfunction in society	
Lack of information about sexual dysfunction associated with prostate cancer therapy in the media.	
No consultant is available to advise about sexual dysfunction, and the topic is not raised even in the prostate cancer patient gathering.	
2) Inadequate information on selecting treatments causes distress among men	
When deciding on a treatment method, men are given no explanation about the effects of a specific therapy and its impact on sexual function. While providing explanations, doctors do not share information clearly about sexual dysfunction and sex life after the treatment.	
Men worry, because they cannot imagine having sexual function and a sex life after treatment, but choose a treatment only to stay alive.	
Men opt for a treatment without understanding the resulting sexual dysfunction or without knowing its impact on sexual function. They feel distressed when they face post-treatment sexual dysfunction.	
3) Hoping for information that promotes an accurate understanding of sexual dysfunction before deciding on treatment methods	
Men expect information in clear, unambiguous words and through media, which would facilitate an accurate understanding of the sexual dysfunction associated with therapy so that they can accept it and move forward in prostate cancer treatment.	
Men expect information on the suitability of various prostate cancer treatments, details of possible sexual dysfunction, the probability of its occurrence, prognostic prospects, and the likelihood of recovery.	
4) Expectations for information on coping with sexual dysfunction	
Men desire information on erectile treatment and reproductive medicine, ways to get erectile aids and orgasms, and how to deal with marital issues.	
3. Need for professional care for individuals and couples with sexual dysfunction	
1) Change in intimate relationship with a partner	
The partner does not understand the patient’s suffering from the loss of sexual function, and the relationship disintegrates.	
The relationship ends because of a partner’s lack of acceptance of a sexless relationship.	
The couple agonizes over having to abandon their sex lives.	
2) Expectations for professional involvement in dealing with the worries of sexual dysfunction	
Men expect consultations from specialists who can deal with diverse sexual dysfunction problems.	
3) Expectations from expert guides to help couples solve their sexual problems.	
Men look to expert guides to help them resolve prostate cancer sexual dysfunction that affects their marital sex life.	
Expectations for an expert guide to help couples solve their sex life problems in line with how they have solved things in the past.	
4. Need for an environment that facilitates interaction among men to solve sexual dysfunction issues	
1) The loneliness of carrying the burden of sexual dysfunction alone	
Men think they have no choice but deal with the obstacles in their sex life because they cannot talk about it publicly.	
Men do not expect medical staff to improve their sexual dysfunction.	
2) Expectations for an environment that facilitates interaction among men and lets them recognize shared sexual dysfunction issues	
Men wish for an environment that facilitates casual interactions and helps them talk about their fear of sexual dysfunction, sort out their feelings, listen to other men about their experiences, and recognize that they have similar issues.	
Men hope to get a chance to interact and talk about sex casually under strict privacy.	

### Need for empathy from medical staff regarding fear of SD

Men commonly perceived sexual functionality to be an important condition to be a “man” and maintain their gender identity within the Japanese socio-cultural context; thus, they expressed their need for medical doctors to provide care for their SD-related identity crisis following PC treatment. However, medical doctors who provide PC treatment in Japanese hospitals are commonly careless about patients’ gender identity crises; they focus on curing cancer and extending life and tend to neglect their patients’ other needs.

I cried when the doctor told me that the treatment would make it impossible for me to have children. I could not ask the doctor any more questions because he said the treatment would cure my cancer. He indicated that life extension, not sex, should be the top priority for cancer patients. (EBRT)

Some medical doctors even make harmful comments regarding patients’ sexual functionality, showing disrespect for their gender identity.

When I searched for the effects of CAB on sexual function on the Internet and told my doctor about it, he said, “At your age, you don’t need it.” Medical institutions are responsible for curing [the] body. But when it comes to issues between the body and mind, they say, “Don’t talk to us because we are too busy.” So even if I wanted to ask questions about SD, I couldn’t. (CAB)

A serious gap exists between medical doctors’ priorities and patients’ needs, which increases patients’ suffering.

Nurses could be an alternative path for patients in seeking mental care and support; however, this route is also restricted because of the gender-related context; men-to-women sexual conversation may be perceived by women as sexual harassment. Since nurses are usually women, male patients fear talking to nurses about their SD gender identity crisis.

I hesitate to discuss sexual problems with nurses, mostly female. I fear they might take it as sexual harassment and despise me. (Prostatectomy)

Patients need care for their SD-related gender identity crisis; however, healthcare professionals’ limited knowledge of patient needs and mental health, their disrespectful attitudes, and the taboo nature of men-to-women sexual conversation are barriers to gender identity crisis care. To remove these barriers, participants insisted that healthcare professionals increase their awareness of patients’ gender identity issues and be willing to discuss patients’ sexual life and help them improve its quality.

I would like nurses to honor the patient’s values when they talk about sexual concerns. (CAB)

Healthcare providers should ask, “How do you feel when you have sex? What do you do when you have trouble getting an erection?” I would be more likely to share my concerns if they asked me, “What are you feeling during sex?” (Prostatectomy)

### Need for information that provides an accurate understanding of SD and coping strategies before deciding on treatments

Participants identified a need for information; they recognized that to overcome an SD-related identity crisis, they must decide on treatment methods based on an accurate understanding of treatment-induced SD and how to manage it. However, in Japan, this type of information is not always provided to patients, who must choose from multiple treatment options. The information provided by medical institutions is biased toward treatments that the institution specializes in, since it is left to the discretion of the physician. Detailed explanations of all treatment options are not provided, which forces patients to choose treatments without knowing how each will affect their sexual function.

It would be good if the doctor explained the pros and cons of surgery, external-beam radiation, and brachytherapy when selecting treatment. (Prostatectomy)

Additionally, healthcare providers sometimes avoid negative language and offer euphemisms, which may prevent patients from accurately assessing their SD risk before treatment, making treatment decisions more difficult. This may even cause patients definitive distress from which they may not recover after treatment.

Before the surgery, the doctor explained that I would no longer be able to have children. After the surgery, I understood the doctor meant that I would not be able to get an erection, have sperm, or have children because I would become impotent. I suffered so much that I considered seeing a psychiatrist. (Prostatectomy)

Before the treatment, the doctor explained to me that it was the same as castration, but I had no idea what that meant. I was very worried that I would never be able to get an erection or ejaculate again and might lose my sex life, an important part of my daily life. Castration is a term used for dogs and cats. I was treated like an animal and could not ask the doctor anything. I tried to convince myself that, to move forward, I needed to be prepared to never have sex again and that I would not need my sexual function once my cancer was cured! (LDR)

Further, in Japanese society, sexuality is focused solely on reproduction; this fosters indifference regarding SD in the elderly, who constitute the majority of PC patients. Consultation services and resources for patients seeking information outside of medical institutions are overwhelmingly lacking.

There are few books on PC. I can only get information from the Internet. Only one Internet bulletin board focused on SD. I tried talking openly about my illness to anyone. But I could not get the necessary information about SD. (LDR)

Thus, patients are left with no information on how to cope with SD after treatment.

I want to know about medications and surgery to treat SD and their costs. (Prostatectomy)

I like to know whether there are any good ways to deal with marital dysfunction and other marital problems. (CAB)

Patients know that accurate information before treatment will help them determine the best treatment for their needs. However, the reproduction-oriented Japanese view of sexuality, physician discretion, and deductive information lead to treatment decisions that patients regret. To remove these barriers, participants argued that they need information that promotes an accurate understanding of SD and potential coping strategies before making treatment decisions.

I think the doctor needs to explain in clear terms before treatment, “If you have surgery, you will become impotent and will no longer be able to have sex.” Then patients will understand that they will not be able to have sex. That way they can work on adapting to their changed bodies after treatment. (Prostatectomy)

### Need for professional care for individuals and couples with SD

Participants’ SD-related identity crises were exacerbated by partners who did not understand the distress of SD and the change in intimate relationships owing to the loss of intercourse, which requires professional care.

My wife did not understand my desire to preserve my sexual function, and our marriage became a wake-up call. With professional intervention, I believe I would have been able to move forward in my relationship with my wife, including deciding on a course of treatment. (Prostatectomy)

My girlfriend told me that she didn’t want a man who couldn’t get an erection, and I was too shocked to talk to anyone about it, so I gave up on remarriage (CAB).

Men sought support in the hope that they and their partner could adapt to changes in their sexual life by applying coping strategies they had developed in their previous joint lives.

Each treatment produces distinct symptoms. Each person has different values. Sexuality is personal. Each patient’s relationship with his wife is also unique. Each couple has built up individual coping strategies over the years. Those strategies deserve respect. (EBRT)

### Need for an environment that facilitates interaction among men to resolve SD issues

Participants stated that they could not publicly discuss their SD nor could they expect help from medical professionals, leaving them with no solution for various related conflicts.

Unlike visual or hearing impairments, SD is a taboo subject and must be resolved in private. (LDR)

Even medical professionals cannot solve an individual’s sexual problems. I must manage my sexual functioning problems on my own. (LDR)

These experiences have also revealed the need for a place to feel private and safe to talk about their concerns, sort out their feelings, and interact with others with the same problems, to gain insight from others’ experiences.

I think it would be good to have a place where people can discuss their sexual issues. It will help them sort out their feelings by speaking out. They hear other people’s opinions and realize that they share similar feelings. (CAB)

I think we need a place where people can exchange opinions anonymously without experiencing abuse, harassment, or irresponsible behavior. (Prostatectomy)

## Discussion

This study explored care needs for SD associated with PC treatment among Japanese men in the gender-related socio-cultural context of Japan, where sexual functionality is a key constituent of male identity, but sexual topics are taboo, even in healthcare settings. Participants identified four care needs from their experiences, summarized as empathy from medical staff regarding SD fears; accurate information to facilitate treatment decisions; professional care for individuals and couples with SD; and an environment that facilitates interaction among men to resolve SD issues. Discussing these needs can help caregivers understand patient experiences more precisely and develop ways to improve patient care.

### Medical professionals need to empathize with patients’ fear of sexual dysfunction

Participants spoke of medical professionals’ lack of involvement in their suffering from SD. Nursing is an interpersonal process [21]. When nurses and patients recognize each other as unique, empathy for the other emerges, which is enhanced by each person’s desire to understand the other. When a nurse wishes to alleviate the cause of the patient’s suffering, empathy develops into sympathy, which allows the nurse to provide the knowledge-supported skills necessary to involve the person. Healthcare providers are not motivated to understand men’s experiences with SD because talking about sex embarrasses them, and they are biased about older adults’ sexuality, especially older adults with cancer [22]. Healthcare providers need proper education to empathize with their patients’ SD fears and recognize their sexual feelings, values, and behaviors. However, most nursing colleges in Japan do not offer human sexuality courses [23]. Education that is systematized to encourage healthcare professionals to empathize with patients’ SD fears would help achieve this goal.

### Information is crucial to understanding sexual dysfunction and potential coping strategies before deciding on treatments

Participants noted that they lacked the necessary information about SD when deciding on PC treatment. The basic bioethics philosophy, introduced in Japan in the 1980s, advocates for respecting medical recipients’ value judgments. Frye emphasizes that autonomy is an ethical principle in nursing, asserting that the amount of information provided to make an informed choice is an external condition of autonomy [24]. However, in Japan, patients do not receive satisfactory explanations on all treatment options and are only informed of the treatment options that their medical institution specializes in. Patients do not uniformly blame medical institutions for not providing sufficient information; although they feel frustrated with their post-treatment SD, they are forced to acknowledge that their lives have been saved, and they must pay the medical institution for their services. Patients need explanations to visualize their SD and post-treatment life, understand coping strategies, and thoroughly examine treatment options.

### Patients with sexual dysfunction and their partners require professional care

Participants stated that they needed to consult with competent professionals to address their diverse and personal concerns about SD. The lack of SD medical care and information has caused patients to lose intimacy with their partners. Wright et al. [25] noted that therapeutic discussions with family members about their expressed beliefs help them recognize or reaffirm their strengths and find solutions to their suffering. Individual consultation based on therapeutic conversation allows for resolution through the forces nurtured in the couple’s history. Japan has few sex therapists or sex counselors [26]. The government has asserted the need to create new occupations and task forces to support the increasing number of cancer patients [27]. Training sexuality specialists and creating sexuality-related medical teams so that patients and medical professionals can discuss difficult sexuality-related issues may be necessary.

### Patients need an environment that facilitates interaction to address sexual dysfunction issues

Participants experienced loneliness when dealing with SD and needed peer support to help resolve their issues. Peer support helps resolve the sexual needs of men with PC and their partners’ sexual adjustment [28]. However, peer support does not function well in Japan, where public discussion of sexuality is not permitted.

In Japan’s Edo period, sex was a sign of the bond between heaven and earth and was considered beneficial for longevity [29]. The pursuit of sex and pleasure was viewed positively, as seen in customs such as night crawling, young men’s lodgings, girls’ lodgings, and shunga (spring pictures). However, the Meiji government, fearing that this culture would be seen as a national disgrace and that the country would be weakened by the spread of venereal diseases, enacted regulations to control sexual mores. People began to monitor each other, and the view that sex was something to be ashamed of and suppressed spread rapidly. Comprehensive sex education, which was introduced in the 1970s, has not penetrated this barrier; sex education in schools still lags behind. This historical and cultural background may contribute to PC patients’ loneliness in Japan. Comprehensive sexuality education is aimed at achieving health, happiness, and dignity, fostering respectful social and sexual relationships, considering how our choices affect our own and others’ well-being, and understanding and ensuring the protection of our rights throughout life [30]. As education is based on these guidelines’ advances, the Japanese public will begin to understand that everyone has the right to the best possible health, happiness, and well-being regarding sex, including enjoyable, satisfying, and safe sexual experiences.

This understanding will change the perception of sex as something to be ashamed of and suppressed to one in which sex is a human right and should be protected. We believe it will also change the way people think and act and promote exchanges to address the SD issue.

### Reflexivity

Some participants may have remained silent about important sexual issues because the interviewer was a woman and health professional. Our analysis and interpretation may be particularly strict on medical professionals because we are a specialized group of oncology nurses.

## Conclusion

This is the first study to explore the care needs of Japanese men for SD associated with PC treatment in the socio-cultural context of Japan. The findings identified the need for healthcare providers’ empathetic attitude, better information regarding PC treatments and their effects on sexual life, potential coping strategies, professional care for couples, and peer interaction regarding SD. Our study findings may facilitate care strategies tailored to these needs. Comprehensive sex education is needed to fully address patient care needs.

## Data Availability

Not applicable.
